# Usual Interstitial Pneumonia in a Patient With Celiac Disease: Expanding the Spectrum of Pulmonary Manifestations

**DOI:** 10.1002/rcr2.70657

**Published:** 2026-06-26

**Authors:** Ragini Gopagoni, Hafsa Fayyaz, Henry Tazelaar, Sushilkumar Sonavane, Prasanth Balasubramanian, Murli Krishna, Remzi Bag

**Affiliations:** ^1^ Department of Lung Transplant Mayo Clinic Jacksonville Florida USA; ^2^ Department of Laboratory Medicine and Pathology Mayo Clinic Scottsdale Arizona USA; ^3^ Department of Cardiothoracic Radiology Mayo Clinic Jacksonville Florida USA; ^4^ Department of Laboratory Medicine and Pathology Mayo Clinic Jacksonville Florida USA

**Keywords:** celiac disease, interstitial lung disease, Lane–Hamilton syndrome, pulmonary fibrosis, usual interstitial pneumonia

## Abstract

Celiac disease (CD) is an autoimmune enteropathy with recognized extraintestinal manifestations. We describe a 55‐year‐old man with long‐standing, well‐controlled CD who developed progressive shortness of breath and cough. Imaging was indeterminate for usual interstitial pneumonia (UIP), and a surgical lung biopsy demonstrated a UIP pattern. Autoimmune serologies were negative, and no alternate etiologies for UIP were identified. With intermittent adherence to a gluten‐free diet and medical management including nintedanib, his disease progressed, ultimately leading to lung transplantation. This case raises the possibility of an association between CD and UIP of uncertain aetiology and should prompt further discussion as the relationship remains unclear. To contextualize this observation, we conducted a systematic review of published case reports evaluating pulmonary manifestations of celiac disease.

## Graphical abstract

This case highlights a rare association between usual interstitial pneumonia and celiac disease, broadening the recognized spectrum of pulmonary involvement beyond Lane–Hamilton syndrome. It emphasizes the importance of considering celiac disease in the evaluation of fibrosing interstitial lung disease, particularly when gastrointestinal symptoms are present.

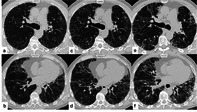

## 1 Introduction

In genetically susceptible individuals, gluten ingestion can lead to Celiac Disease (CD)‐ an autoimmune‐mediated enteropathy with both gastrointestinal and extraintestinal manifestations. Gastrointestinal (GI) manifestations include diarrhoea, malabsorption after gluten ingestion, as well as villous atrophy. Some of the common extraintestinal manifestations include dermatitis herpetiformis, iron deficiency anaemia, weight loss, growth failure in children, peripheral neuropathy, mood disorders, and osteomalacia/rickets [1, 2]. Pulmonary manifestations of CD, however, remain uncommon and rarely described in the literature [3, 4].

An often‐cited pulmonary association of CD is Lane–Hamilton syndrome (LHS), which is rare and defined by the co‐occurrence of idiopathic pulmonary hemosiderosis (IPH) with CD, usually in children [4, 5]. On the other hand, the association of CD with interstitial lung disease (ILD), particularly chronic fibrosing phenotypes like usual interstitial pneumonia (UIP), is not clearly established in the literature and is limited to some case reports [3].

We present a case of a man in his mid‐50s with biopsy‐proven CD on an intermittent gluten‐free diet who developed a progressively fibrosing ILD classified on histopathology as UIP. This case highlights a possible novel pulmonary manifestation of CD, distinct from usual LHS, and stresses the need to further explore the role of the gut‐lung axis in fibrosing lung disease of uncertain aetiology.

To contextualize our observation, we conducted a systematic review in accordance with the Preferred Reporting Items for Systematic Reviews and Meta Analyses (PRISMA) guidelines. PubMed and Scopus were searched using title, abstract, and keyword fields with Boolean combinations of terms related to CD, pulmonary involvement, IPH (LHS), and ILD, including UIP. The search was limited to publications published between 1970 and 2026. Records retrieved from PubMed and Scopus were imported into a single reference library and deduplicated using DOI and title matching, with author name and publication year used when necessary. The number of duplicates removed was recorded and reported in the PRISMA flow diagram. Only case reports involving adult patients (> 18 years) and published in the English language were included.

## 2 Case Report

A 55‐year‐old man with long‐standing CD, diagnosed 19 years earlier based on weight loss, GI symptoms, positive serologic testing and villous atrophy on duodenal biopsy, presented to our clinic for lung transplant evaluation. His disease had been well controlled on an intermittent gluten‐free diet.

His history was also significant for a small sliding hiatal hernia, longstanding gastroesophageal reflux to the thoracic inlet, and episodes of throat clearing and coughing after meals, raising concern for micro‐aspiration. Other comorbidities included obstructive sleep apnea with inconsistent continuous positive airway pressure (CPAP) use, hypothyroidism, migraines, and a remote cerebrovascular accident with complete recovery. Three years prior to the presentation, he began experiencing a gradually worsening dry cough and progressive shortness of breath. He had mild COVID‐19 that was of short duration and was managed in the outpatient setting conservatively. He had occupational exposure to construction dust during a renovation project for 3 months where he wore an appropriate respirator. He did not have any signs or symptoms of telomeropathy. He has never smoked and had no history of substance use. Family history was negative for IPF.

The patient reported episodic musculoskeletal pain involving the knees, hips, and knuckles, attributed to degenerative joint disease. Further evaluation found no clinical or serologic evidence of an autoimmune rheumatologic disorder, with unrevealing inflammatory and extensive autoimmune testing.

Findings on high‐resolution computed tomography (HRCT) scan of the chest were “indeterminate for UIP” or ‘alternate diagnosis’ with possibilities of fibrotic autoimmune/connective tissue disease‐associated ILD, fibrotic organizing pneumonia, or fibrotic hypersensitivity pneumonitis (Figures 1 and 2). On serial comparative CT imaging, mild progression of ILD was seen over 2 years (Figure 3).

**FIGURE 1:**
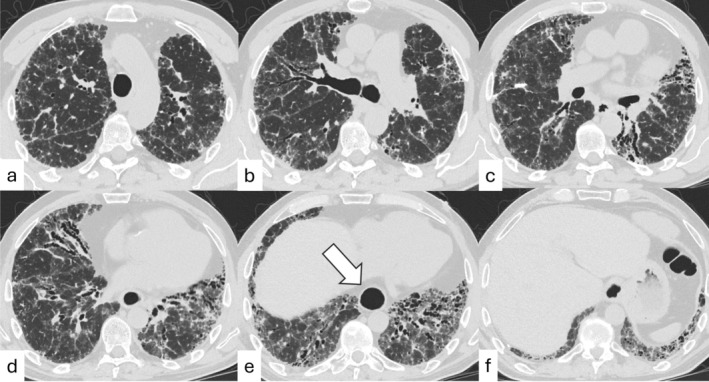
Axial High resolution computed tomography (HRCT) Chest images in inspiration (a, b, c, d, e, f) show mid, lower lung zone predominant, peribronchial and peri lobular distribution patchy ground‐glass, mosaic attenuation, reticulations and traction bronchiectasis. No pulmonary nodules, cysts, or honeycombing. Mild dilation of lower oesophagus is noted (arrow).

**FIGURE 2:**
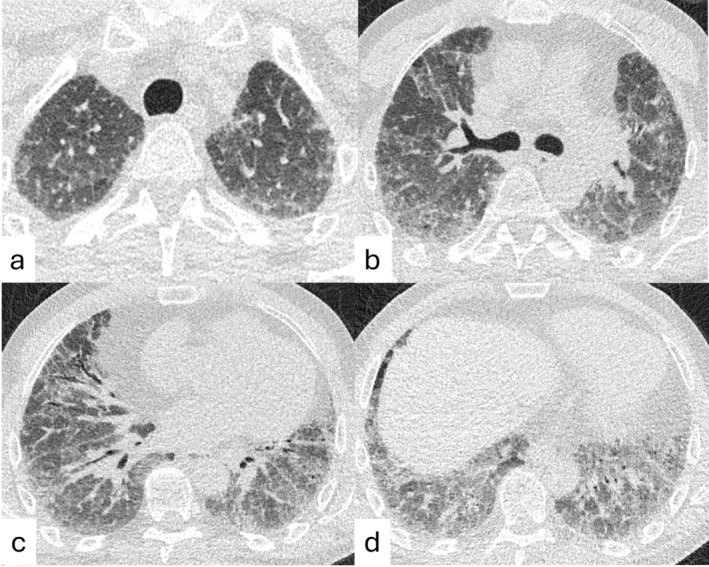
Axial end expiratory images (a, b, c, d) do not show significant air trapping.

**FIGURE 3:**
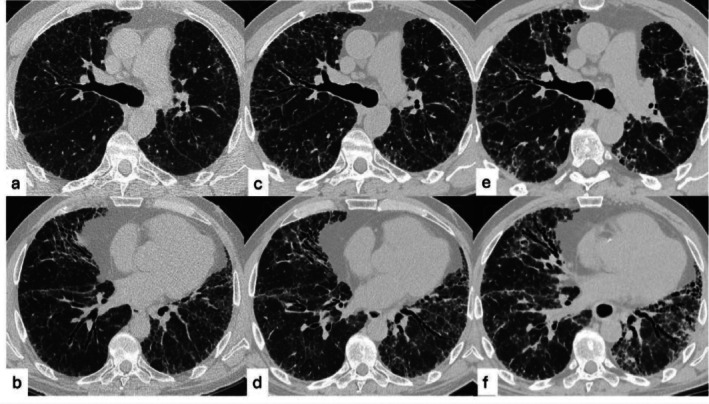
Serial chest CT images at corresponding upper and lower lung zones from 2‐years prior (a, b); 1‐year prior to the presentation (c, d) and the current exam at presentation to the clinic (e, f) showing progression of chronic fibrosing interstitial disease.

Surgical lung biopsy obtained prior to referral to our centre demonstrated UIP pattern, characterized by peripheral and para‐septal fibrosis with fibroblast foci and mild chronic interstitial inflammation, with only rare multinucleated giant cells associated with inspissated mucus. There was no evidence of honeycombing, hemosiderosis, or alveolar haemorrhage.

The case was discussed at multidisciplinary meeting (MDM). Given the patient's never‐smoker status, relatively young age for IPF, with no significant environmental exposures, no autoimmune or connective tissue disease or other features suggestive of telomeropathy, negative family history for IPF, and longstanding CD led the multidisciplinary team to favour a secondary UIP pattern rather than IPF. CD‐associated pulmonary involvement was considered, including a late presenting manifestation within the LHS spectrum or possibly a distinct non‐hemorrhagic pulmonary manifestation with UIP pattern.

Despite medical management with nintedanib, the patient experienced progressive hypoxic respiratory failure, requiring 4 L/min of supplemental oxygen continuously. Serial pulmonary function testing demonstrated a progressively restrictive ventilatory defect with persistently reduced diffusion capacity and interval decline in forced vital capacity (FVC) prior to transplantation (Table 1) (Figure 4). He subsequently underwent successful bilateral lung transplantation.

**TABLE 1:** Serial pulmonary function tests.

Timeline (MM/YY)	FVC (L) (% pred)	FEV1 (L) (% pred)	FEV1/FVC (%)	TLC (L) (% pred)	DLCO ml/(min × mmHg) (% pred)
Baseline (08/23)	2.70 (61.7)	2.48 (71.7)	91.62	—	—
Transplant evaluation baseline (04/24)	2.35 (53.9)	2.20 (64.1)	93.50	3.46 (48.7)	10.31 (37.2)
Prior to transplant listing (02/25)	1.79 (41.2)	1.51 (44.4)	84.57	2.83 (39.9)	9.63 (34.9)

Abbreviations: DLCO, diffusing capacity of the lung for carbon monoxide; FVC, forced vital capacity; FEV1, forced expiratory volume in one second; FEV1/FVC (%), ratio of FEV1 to FVC; L, litre; TLCO, total lung capacity; % pred, percentage predicted.

**FIGURE 4:**
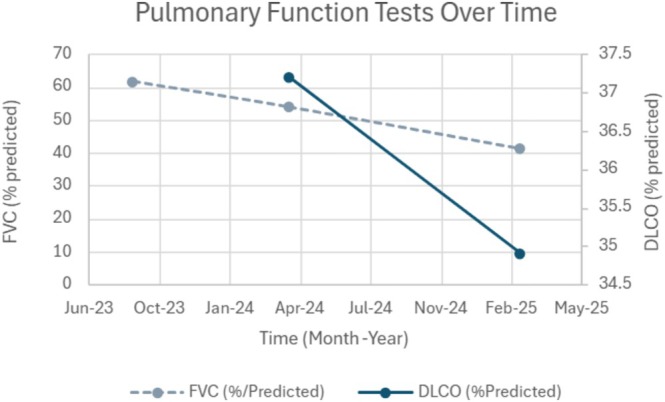
Longitudinal trend of forced vital capacity (FVC) and diffusion capacity for carbon monoxide (DLCO) expressed as percent predicted, demonstrating progressive decline prior to lung transplantation.

## 3 Discussion

This case highlights a diagnostically complex presentation of progressive fibrosing ILD in a patient with biopsy‐confirmed CD, raising possibility of an association of UIP as a pulmonary manifestation of gluten‐sensitive enteropathy. Although CD is primarily recognized as a GI disorder, there are a variety of associated pulmonary manifestations reported in literature, IPH being the most common. Histologic evaluation from this patient revealed a UIP pattern with imaging suggesting ‘indeterminate for UIP’ or ‘alternate diagnosis’. The discrepancy between imaging and histopathology observed in our case is not uncommon in ILD diagnostics reinforcing the importance of a multidisciplinary discussion involving clinicians, radiologists, and pathologists to establish an accurate diagnosis [6].

Recurrent hemoptysis, dyspnea, cough, and iron‐deficiency anaemia are among the most commonly reported symptoms in LHS, with some patients progressing to hypoxemia and respiratory failure. Notable patterns in LHS include the co‐occurrence of hemoptysis and diarrhoea, irrespective of the age groups. The subtle or absent clinical signs of CD in many IPH cases underscore the importance of routine CD screening for timely recognition of LHS [4]. Our patient did not exhibit classic features of IPH, such as hemoptysis or iron‐deficiency anaemia. CD has also been associated with obstructive airway symptoms such as asthma and chronic cough [7]. Findings from the systematic review of the literature on pulmonary manifestations associated with CD are comprehensively summarized in Table 2.

**TABLE 2:** Reported pulmonary manifestations associated with Celiac disease.

Author (Year)	Pulmonary diagnosis	Key findings	Treatment	Outcome
Lane and Hamilton (1971) [8]	IPH	Idiopathic steatorrhea with IPH; first description of association	Azathioprine + gluten free diet	Improved
Pacheco et al. (1991) [9]	IPH	BAL + transbronchial lung biopsy: confirmed IPH. Celiac disease was diagnosed subsequently.	Gluten free diet	Improved with gluten free diet
Bouros et al. (1994) [10]	IPH	BAL: hemosiderin laden macrophages. Confirmed celiac disease	Gluten free diet	Improved with gluten free diet
Malhotra et al. (2005) [11]	IPH	BAL + transbronchial lung biopsy: confirmed IPH. Celiac disease was diagnosed subsequently.	Gluten free diet	Improved with gluten free diet
Agarwal et al. (2006) [12]	IPH	BAL + transbronchial lung biopsy: confirmed IPH. Confirmed celiac disease	Gluten free diet	Improved with gluten free diet
Mayes et al. (2008) [13]	IPH	Transbronchial lung biopsy: confirmed IPH. Confirmed celiac disease	Gluten free diet, prednisone, azathioprine	Improved with immunosuppressive agent
Grover et al. (2010) [14]	IPH	Confirmed IPH and Celiac disease. Celiac disease, Epilepsy and Cerebral calcification (CEC) syndrome. Presented with cerebral venous thrombosis.	Gluten free diet, external ventricular drain insertion, anticoagulation	Improved
Nishino et al. (2010) [15]	IPH	Bronchoscopy + Lung biopsy: confirmed IPH Confirmed celiac disease	Not specified	Not specified
Singhal et al. (2013) [16]	IPH	BAL: hemosiderin laden macrophages. Celiac disease was diagnosed subsequently.	Gluten free diet	Improved with gluten free diet
Kolilekas et al. (2014) [17]	UIP	Childhood Celiac disease with polyglandular autoimmune syndrome IIIA HRCT + Lung Biopsy: UIP	Corticosteroids + Gluten free diet + Mycophenolate mofetil	Deceased due to acute exacerbation
Khilnani et al. (2015) [18]	IPH	BAL + transbronchial lung biopsy: confirmed IPH. Celiac disease was diagnosed subsequently. Associated with dilated cardiomyopathy	Gluten free diet, standard therapy for cardiomyopathy	Improved with gluten free diet
Berger et al. (2015) [19]	IPH	Lung biopsy: confirmed IPH and concomitant celiac disease	Gluten free diet, steroids	Improved
Popp et al. (2016) [20]	Pulmonary hemosiderosis	BAL: rare blood cells and numerous siderophages. Celiac disease was diagnosed subsequently	Glucocorticoids + gluten free diet	Improved with gluten free diet and corticosteroid therapy
Austin et al. (2021) [21]	IPH	VATS + lung biopsy: confirmed IPH. Confirmed celiac disease, associated Membranous Nephropathy	Gluten free diet, steroids	Not specified
Mishra et al. (2022) [22]	IPH	BAL + transbronchial lung biopsy: confirmed IPH. Confirmed celiac disease	Gluten free diet, steroids	Improved with gluten free diet
Fontes et al. (2023) [23]	IPH	Bronchoscopy + BAL: alveolar macrophages, confirmed celiac disease, associated Down syndrome	Gluten free diet, corticosteroid	Improved with steroids
Grech et al. (2023) [24]	IPH	Right VATS + wedge biopsy: hemosiderin laden macrophages, lymphocytic bronchiolitis > IPH. Confirmed celiac disease.	Mycophenolate mofetil + Steroids + Gluten free diet	Improved with gluten free diet and immunosuppressive agent
Ghassa (2024) [25]	Bronchiectasis + Chronic rhinosinusitis	CT Chest/BAL/Spirometry confirming bronchiectasis Confirmed celiac disease	Bronchiectasis management + gluten free diet	Improved with gluten free diet, intermittent diarrhoea due to non‐compliance, respiratory symptoms improved
Ragheb et al. (2025) [26]	IPH	Transbronchial biopsy: confirmed IPH. Confirmed celiac disease	Gluten free diet, corticosteroid	Improved

Abbreviations: BAL, bronchoalveolar lavage; B/L, bilateral; HRCT, high resolution computed tomography; IPH, idiopathic pulmonary hemosiderosis; UIP, usual interstitial pneumonia; VATS, video assisted thoracoscopic surgery.

While the patient reported a brief exposure to cement dust, he wore an appropriate respirator at that time and the biopsy showed no features of pneumoconiosis, for example, silicosis. The CT showed no air trapping, and he had no obvious exposures to organic antigens typically associated with hypersensitivity pneumonitis. The absence of systemic autoimmune features and negative serologies ruled out connective tissue disease‐associated ILD.

UIP represents the histopathological pattern of a severe, chronic, and irreversibly progressive pulmonary fibrosis, which can be due to IPF or occur secondary to various conditions, including autoimmune rheumatic diseases, medication exposure, fibrotic hypersensitivity pneumonitis and other environmental exposures [27, 28]. Interestingly, a UIP pattern in the setting of an overlapping autoimmune syndrome has been previously reported in a patient with CD [17]. However, our patient had no other autoimmune conditions, and self‐reported symptoms of his CD were well‐controlled with an intermittent gluten‐free diet. Given these, multidisciplinary discussion raised the possibility of UIP as a novel pulmonary manifestation of CD, distinct from usual LHS, and other pulmonary manifestations of CD including bronchiectasis, lymphocytic bronchoalveolitis, and chronic sinusitis [25, 29]. The pathophysiology linking CD to pulmonary manifestations remains incompletely understood, and may involve a shared immunologic susceptibility to gluten‐derived peptides, proinflammatory cytokines, immune dysregulation and epithelial injury. Our case report stresses the need to further explore the role of the gut‐lung axis in UIP and other fibrosing lung diseases of uncertain aetiology.

## Author Contributions

R.G. and H.F. were involved in data collection, interpretation of clinical findings, and drafting of the manuscript. R.G. was involved in the literature review and critically revised the manuscript. R.B. conceived the study, participated in the direct clinical care and management of the patient, supervised data collection and the systematic literature review, interpreted the clinical findings within the context of the existing literature, critically revised the manuscript for important intellectual content, approved the final version for publication, and serves as the corresponding author. H.T. reviewed and interpreted the histopathologic findings, contributed to the pathology section, and critically revised the manuscript. S.S. reviewed and interpreted the radiographic findings, contributed to the imaging section, and critically revised the manuscript. All authors reviewed and approved the final version of the manuscript and agree to be accountable for all aspects of the work.

## Funding

The authors have nothing to report.

## Ethics Statement

Ethical approval was not required for this case report according to institutional guidelines because all patient information was deidentified.

## Consent

The authors declare that written informed consent was obtained for the publication of this manuscript and accompanying images using the form provided by the Journal.

## Conflicts of Interest

The authors declare no conflicts of interest.

## Data Availability

The data that support the findings of this study are available on request from the corresponding author. The data are not publicly available due to privacy or ethical restrictions.
